# Enhancement of cognitive and neural functions through complex reasoning training: evidence from normal and clinical populations

**DOI:** 10.3389/fnsys.2014.00069

**Published:** 2014-04-28

**Authors:** Sandra B. Chapman, Raksha A. Mudar

**Affiliations:** ^1^Center for BrainHealth®, The University of Texas at DallasDallas, TX, USA; ^2^Department of Speech and Hearing Science, University of Illinois at Urbana-ChampaignChampaign, IL, USA

**Keywords:** cognitive training, gist reasoning, cognition, neural, brain plasticity

## Abstract

Public awareness of cognitive health is fairly recent compared to physical health. Growing evidence suggests that cognitive training offers promise in augmenting cognitive brain performance in normal and clinical populations. Targeting higher-order cognitive functions, such as reasoning in particular, may promote generalized cognitive changes necessary for supporting the complexities of daily life. This data-driven perspective highlights cognitive and brain changes measured in randomized clinical trials that trained gist reasoning strategies in populations ranging from teenagers to healthy older adults, individuals with brain injury to those at-risk for Alzheimer's disease. The evidence presented across studies support the potential for Gist reasoning training to strengthen cognitive performance in trained and untrained domains and to engage more efficient communication across widespread neural networks that support higher-order cognition. The meaningful benefits of Gist training provide compelling motivation to examine optimal dose for sustained benefits as well as to explore additive benefits of meditation, physical exercise, and/or improved sleep in future studies.

## Introduction

Five decades of research have shown that the brain is modifiable in the context of stimulating cognitive experiences and in response to cognitive training (e.g., Bennett et al., 1964; Schooler, 1984; Schooler et al., 1999). Cognitive training involves guided practice on tasks targeting specific cognitive functions (e.g., working memory, attention) or specific cognitive strategies (Clare and Woods, 2004; Martin et al., 2011). In general, evidence from most cognitive training studies reveal improvements in performance, especially in cognitive functions directly targeted by training (Nyberg et al., 2003; Dahlin et al., 2008; Jaeggi et al., 2008; Valenzuela et al., 2008; Carlson et al., 2009; Zelinski, 2009; Anguera et al., 2013; Chapman et al., 2013). However, much remains to be learned about the kinds of training programs that provide meaningful changes beyond the specific skills trained. From a public health perspective, cognitive training will be deemed useful if the training has generalized benefits and builds cognitive capacities to support performance in day-to-day tasks (Anand et al., 2011).

Concerted efforts are being directed toward exploring the realities of augmenting cognitive performance. Memory training has been a key focus of cognitive training programs motivated by extant evidence that declines in memory commonly occur with normal aging and brain diseases/injuries (e.g., Papp et al., 2009; Gates et al., 2011). Memory training mostly yields short-term improvements in memory; however, benefits do not generalize/transfer to other cognitive functions (Papp et al., 2009; Valenzuela and Sachdev, 2009; Martin et al., 2011; Tardif and Simard, 2011; Reijnders et al., 2012; Teixeira et al., 2012; Thompson et al., 2013), nor do they produce long-term benefits (Rebok et al., 2014). Emerging evidence suggests that reasoning training has widespread and lasting benefits that may guard against and restore cognitive losses in aging and/or disease (Willis et al., 2006; Anand et al., 2011; Chapman et al., 2013; Rebok et al., 2014). For instance, in one of the largest randomized cognitive training trials to date, i.e., the Advanced Cognitive Training for Independent and Vital Elderly (ACTIVE) trial, participants who underwent reasoning training (i.e., focused on solving problems related to serial patterns and sequences) showed less decline in self-reported Independent Activities of Daily Living over a 10-year period compared to the memory training group (i.e., strategies to improve verbal episodic memory) (Rebok et al., 2014).

A growing body of evidence suggests that advanced reasoning engages gist-based processing (Reyna and Brainerd, 1995, 2011; Reyna and Lloyd, 2006). In the current paper, we present a perspective on a particular type of reasoning training that targets *gist-based reasoning* abilities. Gist reasoning is defined as the ability to synthesize and create abstract meanings from the literal content/information, a skill essential for academic, occupational, and functional competence. The primary objective of strategy-driven Gist reasoning training is to improve the ability to abstract generalized meanings from complex information and to incorporate these strategies into everyday tasks. Gist training is informed by cognitive theories of discourse meaning structure (Van Dijk et al., 1983) and information processing (Reyna and Brainerd, 1995), specifically van Dijk and Kintsch's macrostructure/global meanings and Reyna and Brainerd's gist representation. In this perspective paper, we provide a general framework of Gist reasoning training and highlight converging findings from Gist reasoning training studies across adolescent and adult populations. This novel strategy-based approach to cognitive training may provide insights and future directions to guide testing and development of training protocols that have ecological validity/real life application.

## General overview of cognitive training protocols

In the distinct studies summarized below, we examined the potential for Gist reasoning training to improve cognitive performance as compared to control protocol/s (active control/wait-list control) (see Table 1) using a pseudo-randomized control design. We present data from studies on two groups of adolescents [i.e., typically developing eighth graders; youth with traumatic brain injury (TBI)] and three adult populations (i.e., adults with TBI, adults with early mild cognitive impairment, and cognitive healthy adults). Overall, the length of the training period across studies was short-term, ranging from 8 to 12 sessions delivered over one to two months in 45 to 60 min. duration, with each study protocol being identical for all participants within the trial. When an active control protocol was used (Memory strategy or New learning), it was comparable to the Gist reasoning training in length, complexity, and active group discussion/engagement.

**Table 1:** **Brief description of experimental and control training protocols**.

**Training**	**Description**
**EXPERIMENTAL TRAINING**	
*Gist Reasoning Training* (Gamino et al., 2010; Anand et al., 2011; Vas et al., 2011; Chapman et al., 2013; Mudar et al., 2013; Motes et al., 2014; Cook et al., under review)	Hierarchical Strategies
	Strategic Attention: Consciously blocking/inhibiting distractions and irrelevant/less relevant information
	Integrated Reasoning: Binding explicit facts with world knowledge to construct generalized/abstracted meanings
	Innovation: Deriving multiple interpretations and generalized applications beyond the concrete content reflecting fluency and fluidity of thinking
**CONTROL TRAINING**	
*Memory Strategy Training* (Gamino et al., 2010; Cook et al., under review)	Training of bottom-up memory strategies: focus on encoding, rehearsal, retrieval practice, cross modality associations and developing mnemonics
*New Learning Training* (Vas et al., 2011; Mudar et al., 2013) (Gamino et al., 2010)	New Learning about brain functions and influences on cognition
	Example topics covered: Brain Structures and Functions; The Neuron; Neuroplasticity and Neurogenesis; Memory and the Brain; Executive Functions of the Brain; Effects of Sleep and Stress on the Brain; Diet and Exercise and the Brain; Social Bonds and the Brain. This program was originally developed at the Rotman Institute, Canada and is referred to as Brain health workshop (Binder et al., 2008).
*Wait-list controls* (Chapman et al., 2013; Motes et al., 2014)	No contact group

## Gist reasoning training

The Gist reasoning training (also referred to as gist training) is a strategy- rather than content-based program. The protocol entails three core strategies: *strategic attention, integrated reasoning*, and *innovation* summarized in Table 1, delineated in a training manual, and defined in more detail elsewhere (e.g., Gamino et al., 2010; Vas et al., 2011; Chapman et al., 2012; Chapman and Mudar, 2013). The strategies facilitate cognitive control and depth of encoding to facilitate knowledge acquisition and creation. The strategy instruction is hierarchical and dynamically interdependent, with each strategy building on previous strategies, and involves practice that encourages integrating all steps when tackling mental activities both inside and outside of training.

## Evidence of gains from gist training vs. control conditions

### Cognitive training in youth (Gamino et al., 2010; Motes et al., 2014; Cook et al., under review)

#### Typically developing adolescents

In this study, middle-school students (8th graders) were randomly assigned to one of three training protocols, either the Gist reasoning, or one of two control groups, i.e., the Memory training or the New learning (Gamino et al., 2010). The trainings were delivered over 9 class periods lasting 45 min over four weeks. These students were from lower SES backgrounds (92% living in poverty). The three groups were comparable in age, gender, memory, and cognitive abilities at baseline. Outcomes comparing pre- to post- training performances were scored by researchers blinded to individuals and group identity.

Results revealed that gist-trained students improved ability to abstract novel meanings from lengthy classroom-type texts. Additionally, these gist-trained students showed significant improvement in memory for facts, a skill not targeted in training. We found a significant relation between gains in ability to abstract meanings and a real life school measure, the Texas Assessment of Knowledge and Skills (TAKS) reading testing “Applying Critical Thinking Skills.” In contrast, students in the Memory training showed improvements only in memory for isolated facts with no gains in abstracting meanings. The New learning group did not show any significant gains. These findings suggest that gist reasoning strategies may have a broader impact on learning that improves deeper understanding of information encountered beyond shallow learning of isolated facts.

In a subsequent study Motes et al. (2014) examined the effects of Gist training on neural mechanisms related to inhibitory control using electroencephalograph (EEG). Participants in the Gist training group vs. a Wait-list control group completed three visual go/no-go tasks that involved varying levels of semantic categorization (basic to more abstract superordinate categorization) both before and after training or a comparable duration in the case of the controls. The findings revealed that participants in the Gist group showed significant improvement in inhibition (i.e., ability to withhold behavioral responses on no-go trials) following training unlike the control group across basic and superordinate categorization tasks. Furthermore, those in the Gist trained group showed EEG changes suggestive of improved processing efficiency, as reflected by significant reduction in P3 no-go amplitude post-training compared to pre-training. No such differences were observed in the Wait-list controls. Overall, both the behavioral findings and the electrophysiological data across these two studies suggest that Gist training appears to enhance inhibitory responses both at the behavioral and neural level in typically developing middle school children. However, these findings have to be further validated using additional procedures (e.g., positive and negative patterning) in future studies.

#### Adolescents with TBI

The benefits of Gist training vs. Memory training in adolescents with TBI (ages 12–20 years) at chronic stages post injury (>6 months) was evaluated in a recent study (Cook et al., under review). Participants received one-on-one training delivered in eight sessions of 45 min duration over a four week period. The text materials for the Memory training were largely the same content as used in the Gist protocol. The findings revealed that the Gist-trained group significantly improved in their ability to abstract/synthesize meanings as compared to the Memory-trained group. The Gist-trained group also showed enhanced performance on cognitive measures for memory for facts, working memory (Digit Span backwards and Letter-Number Sequencing—WAIS III or WISC IV), and inhibition (Color Word Interference—D-KEFS). None of these latter cognitive skills were specifically targeted during training, suggesting spill-over effects of the Gist training to untrained cognitive domains. The Memory trained group's performance on memory for facts approached significance; however, results failed to show significant gains in domains of abstracting meaning, working memory, inhibition, or other cognitive areas.

Taken together, evidence from these three studies implicates the potential to enhance cognitive performance in areas of cognitive control in typically developing teens and teens with TBI, beyond the traditional treatment phase of 3 months post injury. Augmenting cognitive performance in normally developing populations and individuals at chronic stages post brain injury represents newer and promising areas of investigation.

### Adult cognitive training trials (Anand et al., 2011; Vas et al., 2011; Chapman et al., 2013)

#### Adults with TBI

In a randomized trial, the effects of Gist-reasoning training were compared to an active control training involving New learning in adults with TBI (ages 29–65 years) in chronic stages post-injury (>one year) (Vas et al., 2011). The Gist-trained group exhibited significant gains in abstracting meanings from complex information as compared to the New learning training group. Moreover, the Gist-trained group showed significant enhancement on measures of immediate memory (Digit Span Forward—WAIS-III), executive functions of working memory (Letter Number-Sequence—WAIS III and Daneman and Carpenter listening span) and cognitive switching (Color-Word Interference task—D-KEFS), and non-verbal reasoning (Matrix Reasoning—WAIS III). None of these latter cognitive processes were specifically targeted during training, adding further evidence of generalized benefits from Gist training. Furthermore, this group reported significant improvement in daily life skills (GOS-E, Functional Status Examination, Community Integration Questionnaire), such as increased socialization (e.g., initiating and planning activities with family and friends), higher levels of life productivity (e.g., active job seeking, setting up interviews, improved work efficiency and output), improved personal management (e.g., completing household responsibilities and house upkeep) and better sense of overall well-being. These reported real life gains from Gist training are not likely due to placebo effects since both groups believed they were receiving the experimental training. These training-related gains were maintained at 6-month follow-up. Despite comparable levels of active and engaged learning, the New Learning group failed to show significant gains on any of the performance measures.

#### Adults with pre-clinical Alzheimer's (mild cognitive impairment)

This study compared immediate benefits of Gist training vs. New learning on cognition in individuals with Mild Cognitive Impairment (MCI) in a random assignment design (Mudar et al., 2013). Groups were comparable in age, Mini-Mental State Examination (MMSE) and episodic memory scores at baseline. Both groups received 8 h of training over a period of 4 weeks. We found differential cognitive gains between the two groups. Significant improvement was observed in the Gist-trained group on measures of abstract meaning, strategic attention, memory (immediate and delayed recall Logical Memory subtest-WMS III) and abstract verbal reasoning (Similarities-WAIS III). In contrast, participants in the New learning group showed significant improvements in remembering facts on an experimental text memory measure and on the Sorting test (D-KEFS). These findings suggest that both trainings offered some benefit for those with a progressive neurologic disease; however, Gist training offered broader benefits. Given the lack of pharmacological treatment options in slowing cognitive deterioration in pre-clinical stages of dementia, one current focus is on identifying non-pharmacological options (e.g., cognitive training) that can slow the rate of cognitive deterioration. Although maintenance of gains from Gist training needs to be studied in larger trials, even short-term increases in cognitive performance offer a glimmer of hope. Different doses and more frequent time intervals of cognitive training needs to be examined to harness optimal benefits in terms of maintaining cognitive capacity and slowing decline.

#### Cognitively healthy adults

**Figure 1:**
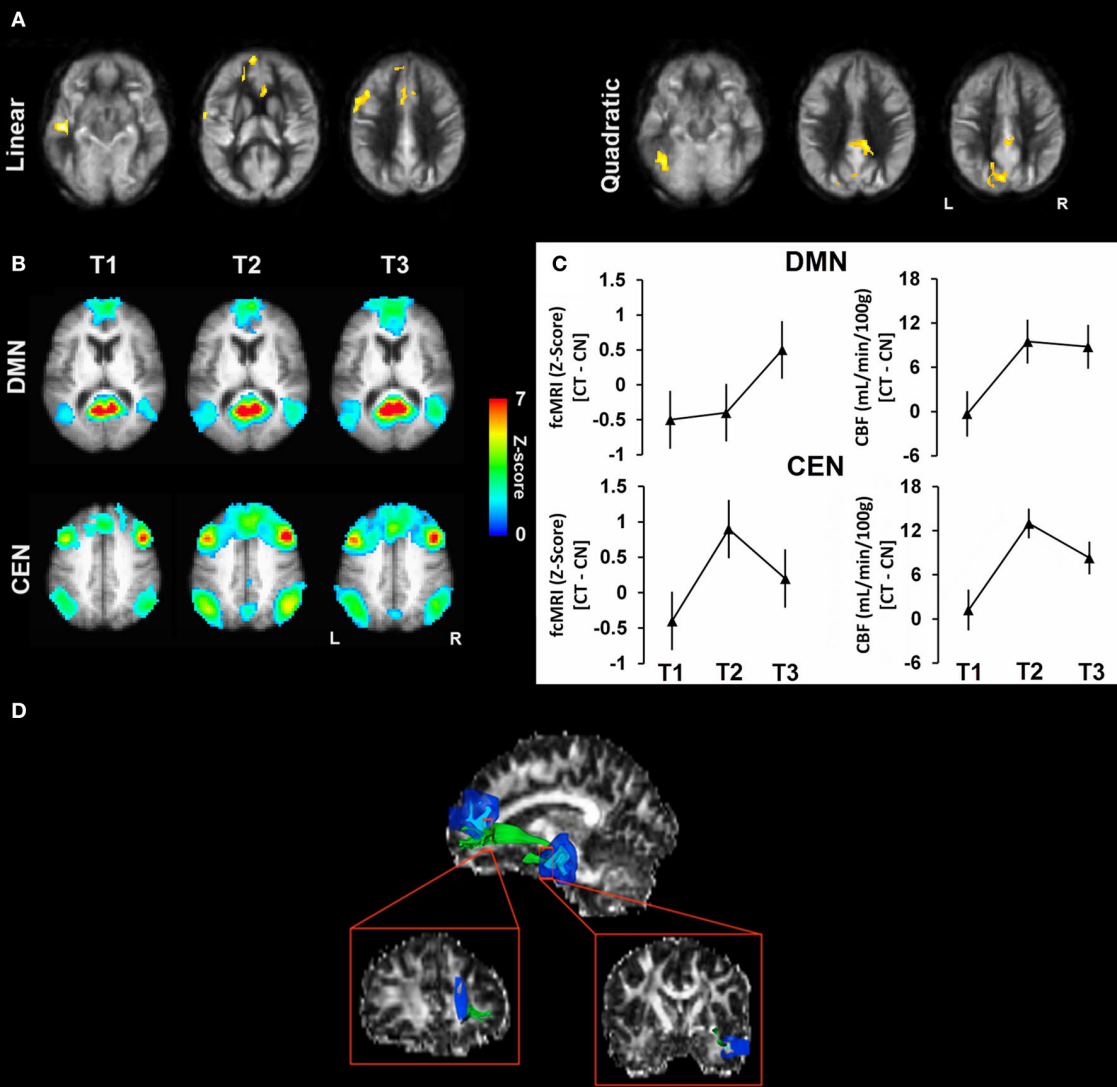
**This figure illustrates the convergence of neural plasticity findings in the cognitive training vs. control group across cerebral blood flow, functional connectivity, and structural DTI changes implicating functional brain changes more frequent and rapid than structural plasticity comparing changes at T2 and T3 to baseline T1 measures. (A)** Results of CBF voxel-based comparison superimposed on an average CBF map of all participants for linear and quadratic interaction contrasts. **(B)** The average functional connectivity maps (i.e., DMN and CEN) of the cognitive training group are overlaid on their average T1-weighted image. **(C)** Mean increase in fcMRI z-scores (left column) and mean change in absolute CBF (right column) are shown for DMN and CEN across time periods. **(D)** A representative participant's uncinate fasciculus (green) is overlaid on his fractional anisotropy map from DTI (Chapman et al., 2013).

The short-term effects of (i.e., 12 weeks of 1-h in-person training/week + 2 h/week homework) Gist training on cognitive and neural plasticity was examined in cognitively healthy adults (55—75 years of age), well-screened to rule out early dementia, as compared to a Wait-list control group (Chapman et al., 2013). Individuals were assessed at baseline, at midpoint and immediately post-training. The Gist-trained group showed generalized cognitive gains consistent with an earlier pilot study in healthy older adults in which participants improved in cognitive switching (Trail Making Test Part B), concept abstraction (Similarities-WAIS III), and fluency (COWAT letter fluency) (Anand et al., 2011). We examined changes in large-scale brain networks in terms of brain blood flow (CBF) and functional and structural connectivity, in addition to the relationship between improved cognitive performance and brain changes. Results revealed significant increases in global CBF (total brain) as well as increased regional CBF measures as measured with Arterial Spin Labeling in two distinct and major brain networks tied to higher-order cognition, namely the Default Mode Network (DMN) and the Central Executive Network (CEN). Specifically, the DMN and CEN have been associated with top-down, cognitive control processes (Bressler and Menon, 2010). We also found corroborating patterns of increased functional connectivity in these very same major brain networks as the increased CBF (Figure 1). Additionally, we found significantly increased structural connectivity as measured with diffusion tensor imaging (DTI) in the left uncinate fasciculus—the white matter tract that connects the middle temporal lobe to the superior medial frontal gyri after 12 weeks of training. The finding of increased white matter integrity in this select brain region perhaps suggests that some degree of atrophy at the level of white matter tracts in healthy aging may be reversible through Gist training. The expansion of the uncinate is intriguing and perhaps suggests that this pathway plays a role in synthesizing new learning—linking the memory center (left middle temporal region) to a brain region that is implicated in abstraction skills (e.g., the left superior medial frontal gyrus).

Whereas a handful of studies have reported cognitive gains and brain changes in response to cognitive training (Nyberg et al., 2003; Boyke et al., 2008; Belleville et al., 2011; Rosen et al., 2011), this study provided some of the first convergent findings across multiple brain imaging platforms (CBF, connectivity, white matter) and cognition supporting the promise of strategy-based top-down cognitive training to enhance brain integrity (Chapman et al., 2013). We acknowledge these findings are preliminary and should be interpreted with caution since the gist training group was compared to a wait-list control group. Nonetheless, the significant relation between brain changes and cognitive improvement provides impetus that the brain gains from gist-training may be possible and warrant further study with active controls.

## Conclusions and future opportunities

This synopsis of key findings across studies in normal and clinical populations indicates Gist reasoning training has the potential to improve cognitive performance beyond skills trained with the likelihood of enhancing underlying neural systems, as well as real life functional abilities. The significant improvements were achieved after relatively short-term training periods. The gains were documented in pseudo-randomly assigned trials comparing the experimental Gist reasoning training to control groups using objective measures.

We postulate that top-down strategy-based cognitive training may yield efficient and easily adoptable methods of mental practice to achieve broad-based benefits. To guide future cognitive training trials, we offer several explanations why Gist reasoning training may augment higher-order executive functions. First, gist reasoning takes advantage of the human brain's preferential bias toward understanding generalized/gist meanings (Bartlett, 1932; Reyna, 1996; Gabrieli, 2004). Extant evidence has demonstrated that while memory for details is lost fairly quickly, memory for global/gist meanings is preserved when delayed recall is examined, whether tested 30 min, one day or even a week later (Bransford and Franks, 1971; Bransford et al., 1972; Mandler and Rabinowitz, 1981; Reyna, 1996; Norman and Schacter, 1997; Kahana and Wingfield, 2000; Gabrieli, 2004). Second, gist reasoning requires an active process of meaning-abstraction where the incoming details are integrated within one's repository of world knowledge by a conscious, controlled manipulation of input into précised ideas (Johnson-Laird, 1983; Van Dijk et al., 1983; Frederksen and Donin, 1991; Zwaan and Radvansky, 1998; Chapman and Mudar, 2013). This integration of incoming data with prior knowledge necessitates activation of top-down processing with enhanced depth of encoding compared to simple representation of literal input. Third, gist reasoning is a practical skill that the majority of people from adolescence to old age can implement and practice throughout their daily normal mental activities (Lloyd and Reyna, 2009; Gamino et al., 2010; Vas et al., 2011; Motes et al., 2014). Examples of gist reasoning are meaning-creations illustrated by generating interpretations, themes, take-home messages, synopses, or generalized statements, to mention a few forms. Fourth, accruing evidence suggests that such a top-down approach to processing engages broad-based brain networks (Gazzaley et al., 2005; Chen et al., 2006, 2011; Chapman et al., 2013). We propose that when the neural activity of major brain networks is increased through complex and meaningful cognitive activities involving gist reasoning, the outcomes may be manifested at multiple levels of cognitive performance and neural health.

Immense potential exists to augment cognitive performance and enhance neural systems through top-down cognitive activity, such as gist reasoning. Future research opportunities should combine multiple approaches simultaneously from the growing armamentaria shown to enhance cognitive performance and brain functions to examine additive benefits. The current reported benefits from Gist reasoning training motivate future trials where the gist training protocol is combined with methods such as short-term meditation (Tang et al., 2007), sensory processing training (Mahncke et al., 2006), aerobic exercise (Kramer and Erickson, 2007; Chapman et al., 2013), or protocols to improve sleep (Nedergaard, 2013). Also critical is examining subjects' motivational level as a factor. Expanded efforts to identify combinatorial protocols that strengthen cognitive performance and recoup losses will be of major public health significance, with the ultimate goal to more fully harness the brain's capacity to be strengthened in health, disease, and injury.

## Funding

This work was supported by grant from the National Institute of Health (RC1-AG035954, R01-NS067015, R01-AG033106, NICHD R21-HD062835), Department of Defense (W81XWH-11-2-0194) and by grants from the T. Boone Pickens Foundation, the RGK foundation, the Lyda Hill Foundation, Dallas Foundation, and Dee Wyly Distinguished University Endowment.

### Conflict of interest statement

The authors declare that the research was conducted in the absence of any commercial or financial relationships that could be construed as a potential conflict of interest.
